# The differentiation of preadipocytes and gene expression related to adipogenesis in ducks (Anas platyrhynchos)

**DOI:** 10.1371/journal.pone.0196371

**Published:** 2018-05-17

**Authors:** Shasha Wang, Yang Zhang, Qi Xu, Xiaoya Yuan, Wangcheng Dai, Xiaokun Shen, Zhixiu Wang, Guobin Chang, Zhiquan Wang, Guohong Chen

**Affiliations:** 1 Key Laboratory of Animal Genetics and Breeding and Molecular Design of Jiangsu Province, College of Animal Science and Technology, Yangzhou University, Yangzhou, Jiangsu, China; 2 Waterfowl Institute of Zhenjiang City, Dantu, China; 3 Department of Agricultural, Food and Nutritional Science, University of Alberta, Edmonton, Alberta, Canada; Qingdao Agricultural University, CHINA

## Abstract

Meat quality is closely related to adipose tissues in ducks, and adipogenesis is controlled by a complex network of transcription factors tightly acting at different stages of differentiation especially in ducks. The aim of this study was to establish the preadipocyte in vitro culture system and understand the biological characteristics of expansion of duck adipocyte tissue at the cellular and molecular level. We isolated pre-adipocytes from the subcutaneous fat of three breeds of duck and differentiated them into mature adipocytes using a mixture of insulin, rosiglitazone, dexamethasone, 3-isobutyl-1-methylxanthine, and oleic acid over 0,2, 4, 6, and 8 days. Successful differentiation was confirmed from the development of lipid droplets and their response to Oil Red O, and increasing numbers of lipid droplets were stained red over time. The expression of key marker genes, including peroxisome proliferator activated receptor γ (*PPARγ*), *CCAAT/*enhancer binding protein-*α* (*C/EBPα*), adipocyte fatty acid binding protein 4 (*FABP4*), and fatty acid synthetase (*FAS*), gradually increased during pre-adipocyte differentiation. Furthermore, it was verified by interference experiments that the knockdown of *PPARγ* directly reduced lipid production. Meanwhile we analyzed the role of unsaturated fatty acids in the production of poultry fat using different concentrations of oleic acid and found that lipid droplet deposition was highest when the concentration of oleic acid was 300 μM. We also compared the level of differentiated pre-adipocytes that were isolated from Jianchang ducks (fatty-meat duck), Cherry Valley ducks (lean-meat duck) and White-crested ducks (egg-producing duck). The proliferation and differentiation rate of pre-adipocytes derived from Jianchang ducks was higher than that of White-crested ducks. These results provide the foundation for further research into waterfowl adipogenesis.

## Introduction

Anatine adipose tissue is directly associated with the production and meat quality characteristics. It is composed of a large number of clustered adipocytes. And the adipocytes aggregated into clusters are divided into small lobes by thin layers of loose connective tissue. All known adipocytes derive from pluripotent mesenchymal stem cells (MSC) in a process called adipogenesis [1–3]. The proliferation and differentiation of pre-adipocytes leads to the formation of adipose tissue. [4].

Previous studies had shown that the differentiation and adipogenesis in preadipocytes was a very complex process involving a large amount of gene expression.Understanding the molecular mechanism of adipogenesis can help to improve the quality of meat, increase the utilization rate of feed, and contribute to the feed conversion ratio (FCR). Transcriptional factors including CCAAT/enhancer-binding protein *(C/EBP*) family including *C/EBPα*, *C/EBPβ*, and *C/EBPδ* [5,6], and peroxisome proliferator-activated receptor (*PPAR*) [7,8], as well as other genes, such as fatty acid-binding protein (*FABP4*) [9], lipoprotein-lipase (*LPL*), and fatty acid synthase (*FAS*) [10] both played important roles in the formation of fat. *DLK1* (delta drosophila homolog-like 1 or delta-like 1 homologue) first discovered in the neuroblastoma could inhibit the differentiation of pre-adipocytes and was known as pre-adipocyte factor-1 (*pref-1*)[11].

Unlike mammals, adipose tissue in poultry cannot synthesize fatty acids, so that oleic acid is added to the culture medium of dexamethasone, insulin, and 3-isobutyl-1-methylxanthine (IBMX) as an exogenous fatty acid to induce pre-adipocyte differentiation. Oleic acid is both an induction factor and a triglyceride synthesis material [12], and can significantly stimulate the expression of several marker genes related to adipocyte differentiation [13]. Ding et al. [14] showed that a 12 h treatment of oleic acid significantly up-regulated the expression of *FAS* pre-adipocytes in duck.

Adipose tissue is a complex, necessary, highly active metabolic and endocrine organ [15] that could store energy as lipids [16]. Adipose tissue could serve as a crucial integrator of energy balance and glucose homeostasis in humans [17]. However, the unlimited expansion of adipose tissue resulted in obesity, which significantly contributes towards type 2 diabetes, hyperlipidemia, hyperglycemia, insulin resistance, chronic inflammation, and atherosclerosis and so on [18,19]. Therefore, the study of adipose tissue is of great importance for health.

In this study, we demonstrate changes in the expression profiles of adipogenesis-related genes during adipocyte differentiation in three breeds of duck. And we also investigated the role of oleic acid, genetic background, and *PPARγ* in adipogenesis in ducks. These results provide a foundation for further research into the mechanisms of duck adipogenesis.

## Materials and methods

### Ethics statement

All animal experimental procedures were approved and guided by the Institutional Animal Care and Use Committee of the School of Animal Science and Technology, Yangzhou University (Permit Number: 45, Government of Jiangsu Province, China) and the U.S. National Institute of Health Guidelines (NIH Pub. No. 85–23, revised 1996).

### Chemicals

Phosphate buffered saline (PBS) (Hyclone, USA), penicillin/streptomycin (Gibco, USA), 0.1% collagenase type Ι (Gibco, USA), Dulbecco’s Modified Eagle’s medium (DMEM) (Hyclone, USA), fetal bovine serum (FBS) (Gibco, USA), Red Blood Cell Lysis Buffer (Solarbio, China), 0.25% Trypsin-EDTA (Gibco, USA), 3-isobutyl-1-methylxanthine (IBMX) (Sigma, USA), Insulin (Sigma, USA), dexamethasone (Sigma, USA), Rosiglitazone (Sigma, USA), Oleic acid (Sigma, USA), Triton X-100 (Solarbio, China), TRIzol (Life, USA).

### Primary culture of pre-adipocytes from subcutaneous adipose tissue

Pre-adipocytes from Cherry Valley duck (lean-meat duck), White-crested duck (egg-laying duck), and Jianchang duck (fatty-meat duck) were isolated as described below [3]. Cherry Valley duck and Jianchang duck were provided by Jiangsu Yike Food Co., Ltd, and White-crested duck was provided by Waterfowl Institute of Zhenjiang City. Briefly, we separated subcutaneous adipose tissue from 20-day-old embryos using sterile scissors and tweezers and placed it into vial bottles. Adipose tissues were minced into 1-mm^3^ sections using scissors and digested with 2 mg/mL type I collagenase in PBS at 37°C in a shaking water bath for 90 min. The resulting mixture was filtered through 0.300 mm, 0.0750 mm, and 0.0374 mm sieve screens to remove undigested tissue and large cell aggregates after blending with 10% FBS in DMEM. The filtered suspensions were then centrifuged at 1000 × g for 10 min at room temperature to separate floating adipocytes from the pre-adipocytes. The precipitate was resuspended using Red Blood Cell Lysis Buffer for 15 min at room temperature. The pre-adipocytes were seeded into culture flasks and cultured in a humidified atmosphere of 5% CO^2^ and 95% air at 37°C after being centrifuged at 1000 × g for 10 min. The characteristics of pre-adipocytes were observed using a microscope (Olympus, Tokyo, Japan).

### Cell culture and differentiation

Pre-adipocyte were digested using 0.25% Trypsin-EDTA for 1 min after the pre-adipocytes typically formed a large number of colonies and reached 100% confluence at approximately 24 h. Then the pre-adipocyte pellet was washed with PBS, centrifuged at 1000 × g for 5 min, and resuspended with 10% FBS in DMEM, and seeded into 6-well plates and 96-well plates at a density of 1 × 10^4^ cells/cm^2^ with the basic medium. The basic medium was changed to the differentiation medium after achieving 90% cell confluence with 0.5 mmol/L IBMX, 1 μm/L DEX, 10 μg/μL insulin, 1 μM Rosiglitazone, 300 μM oleic acid added to the medium to induce pre-adipocyte differentiation. The medium was replaced every second day. The differentiation medium was replaced with the maintenance medium of 10 μg/μL insulin only after 4 days and cells were incubated for a further 4 days.

### Cell proliferation assays

Pre-adipocytes and differentiated cell proliferation was measured with the Cell Counting Kit-8 (CCK-8, Dojindo Corp, Japan) according to the instructions. Approximately 2,000 cells were transferred into a 96-well plate per well, to which 10 μL CCK-8 reagent was added to 90 μL of the culture medium. The cells were subsequently incubated for 4 h at 37°C when the medium became yellow at 1, 2, 3, 4, 5, and 6 days culturing. Absorbance was measured at 450 nm using a microplate reader to establish the growth curve of cells (n = 5).

### Oil Red O staining

The lipid droplets in pre-adipocytes were stained using an Oil Red O staining kit (Solarbio, China) according to the manufacturer instructions. Briefly, cells were washed using PBS and fixed using 4% formaldehyde for 30 min. Then, the fixed cells were washed using 60% isopropanol for 30 sec and stained with Oil Red O solution (solutions A and B). After 20 min, the cells were rinsed using 60% isopropanol to remove the residual Oil Red O and repeatedly washed using distilled water. Stained cells were observed using a microscope.

### Triglyceride content assay

Triglyceride (TG) content was analyzed using a triacylglycerol detection kit (Jiancheng, China) according to the manufacturer protocols. Briefly, the differentiated pre-adipocytes were washed using PBS and sonicated to ensure they were ruptured. A BCA protein assay kit (Beyotime, Beijing) was used to measure the protein concentration of ruptured cells. The TG content was expressed in mmol/g protein.

### Cloning the full-length coding sequence of *PPARγ*

Total RNA was extracted from Cherry Valley ducks using the RNAsimple Total RNA kit (Tiangen, Beijing, China) according to the manufacturer instructions. First-strand cDNA was synthesized using 1 μg RNA and a PrimeScript II 1st Strand cDNA synthesis Kit (TaKaRa, Dalian, China) according to the manufacturer protocols. Primers were designed using the BGI duck PPARγ sequence (accession: NW_004676594, Table 1). The amplified products were verified by 1.5% agarose gel electrophoresis and purified with a gel extraction kit (Omega, USA). Then, the purified products were ligated into the pMD19-T Vector (TaKaRa, Dalian, China), transferred into E. coli DH5α cells, and sequenced by Sangon (Shanghai, China).

**Table 1:** Primer sequences for PCR.

Gene	Application	Accession num.	Primer	Product size (bp)
*PPARγ*-cds	Clone	NW_004676594	F:CCAAGCTTATGGTTGACACAGAAATGCCG	1428
			R:CGCGGATCCTTAATACAAGTCTTTATAGA	
*PPARγ*	qRT-PCR	NW_004676594	F:TTCCAACTCCCTTATGGC	232
			R:GGCATTGTGTGACATTCC	
*β-actin*	qRT-PCR	EF667345	F:ATGTCGCCCTGGATTTCG	165
			R:CACAGGACTCCATACCCAAGAA	
siPPARγ-54	interference		F:GGAUCUGUCUGCGAUGGAUTT	
			R:AUCCAUCGCAGACAGAUCCTT	
siPPARγ-872	interference		F:GCAGGAGAUCACAGAAUUUTT	
			R:AAAUUCUGUGAUCUCCUGCTT	
siPPARγ-1343	interference		F:GCAGCUGUUGCAAAUAAUATT	
			R:CAUUAUUUGCAACAGCUGCTT	
NC	interference		F:UUCUCCGAACGUGUCACGUTT	
			R:ACGUGACACGUUCGGAGAATT	
NC-FA	interference		F:UUCUCCGAACGUGUCACGUTT	
			R:ACGUGACACGUUCGGAGAATT	
*LPL*	qRT-PCR	NW_004676567	F:ACAATGTCCACTTGCTGG	184
			R:TAGGTGTGTAGGACATCC	
*DLK1*	qRT-PCR	XM_005020731	F:CTGTGCCCTTCTGGTTTTGC	192
			R:TCCATTCTCACATGGGCCAC	
*C/EBPα*	qRT-PCR	KU983766	F:GTGCTTCATGGAGCAAGCCAA	191
			R:TGTCGATGGAGTGCTCGTTCT	
*FABP4*	qRT-PCR	NM_001310375	F:CCCAATGTAACCATCAGC	178
			F: ACTTCTGCACCTGCTTCAG	
*FAS*	qRT-PCR	NW_004676466	R: TCAACCTTCTGCTGAAGC	204
			R: AGCCATCAGTGTTACTCC	

Note: *PPARγ*: peroxisome proliferator activated receptor γ, *LPL*: lipoprotion lipase, *DLK1*: delta-like 1 homologue, *C/EBPα*: *CCAAT/*enhancer binding protein-*α*, *FABP4*: adipocyte fatty acid binding protein 4, *FAS*:fatty acid synthetase. The primer of *CEBP/α*, *FABP4* were picked from Ding et al [3]

### SiRNA knockdown and transfection

Briefly, pre-adipocytes were trypsinized and resuspended in the basal culture medium, then seeded into 24-well plates and transfected with siRNA (GenePharma, Shanghai, China) to target duck *PPARγ* using Lipofectamine 2000 RNAi MAX (Life Technologies) with a serum-free medium at a final concentration of 50 nM. The interference sequence is designed based on the cloned *PPARγ* full-length sequence. A non-sense siRNA (NC) was used as a negative control for non-sequence specific effects. Two days after reaching confluency, cells were induced using the complete induction medium for another 2 days. The sequences of *PPARγ* siRNA were shown in Table 1. Silencing of *PPARγ* was monitored using qRT-PCR.

### Real-time PCR analysis

Total RNA was extracted from cells using TRIzol reagent and the complementary DNA was synthesized from 1 μg of total RNA using reverse transcriptase (Tiangen, China). Quantitative real-time polymerase chain reaction (qRT-PCR) was performed using a Thermo Fisher Scientific (QuantStudio 5) Real-time PCR instrument using SYBR Green qRT-PCR master mix (Vazyme, China). The qRT-PCR reaction consisted of 7.2 μL sterile water, 10 μL SYBR master mix (2 × Low ROX Premixed), 0.4 μL forward primer (10 mM), 0.4 μL reverse primer (10 mM), and 2.0 μL complementary DNA, making a total volume of 20 μL. The parameters of qRT-PCR were as follows: 95°C for 30 s, 40 cycles of 10 s at 95°C, and 30 s at 60°C. The comparative cycle threshold values were used to weight the expression levels of CCAAT/enhancer-binding protein α (*C/EBPα*), peroxisome proliferator-activated receptor γ (*PPARγ*), fatty acid–binding protein 4 (*FABP4*), Lipoprotein Lipase (*LPL*), fatty acid synthase (*FAS*). Fold-change was normalized using the 2^-ΔΔCT^ method, with β-actin as the endogenous control. Each interval included three biological replicates (n = 3). The qRT-PCR primer sequences are summarized in Table 1.

### Statistical analysis

Data were presented as the mean ± standard deviation (SD). Between-group differences were determined using Student’s t test. *P* < 0.05 was considered statistically significant. All statistical analyses were carried out using SPSS (version 17). All experiments were performed in triplicate.

## Results

### Identification of pre-adipocytes and dynamic observations during differentiation

**Fig 1:**
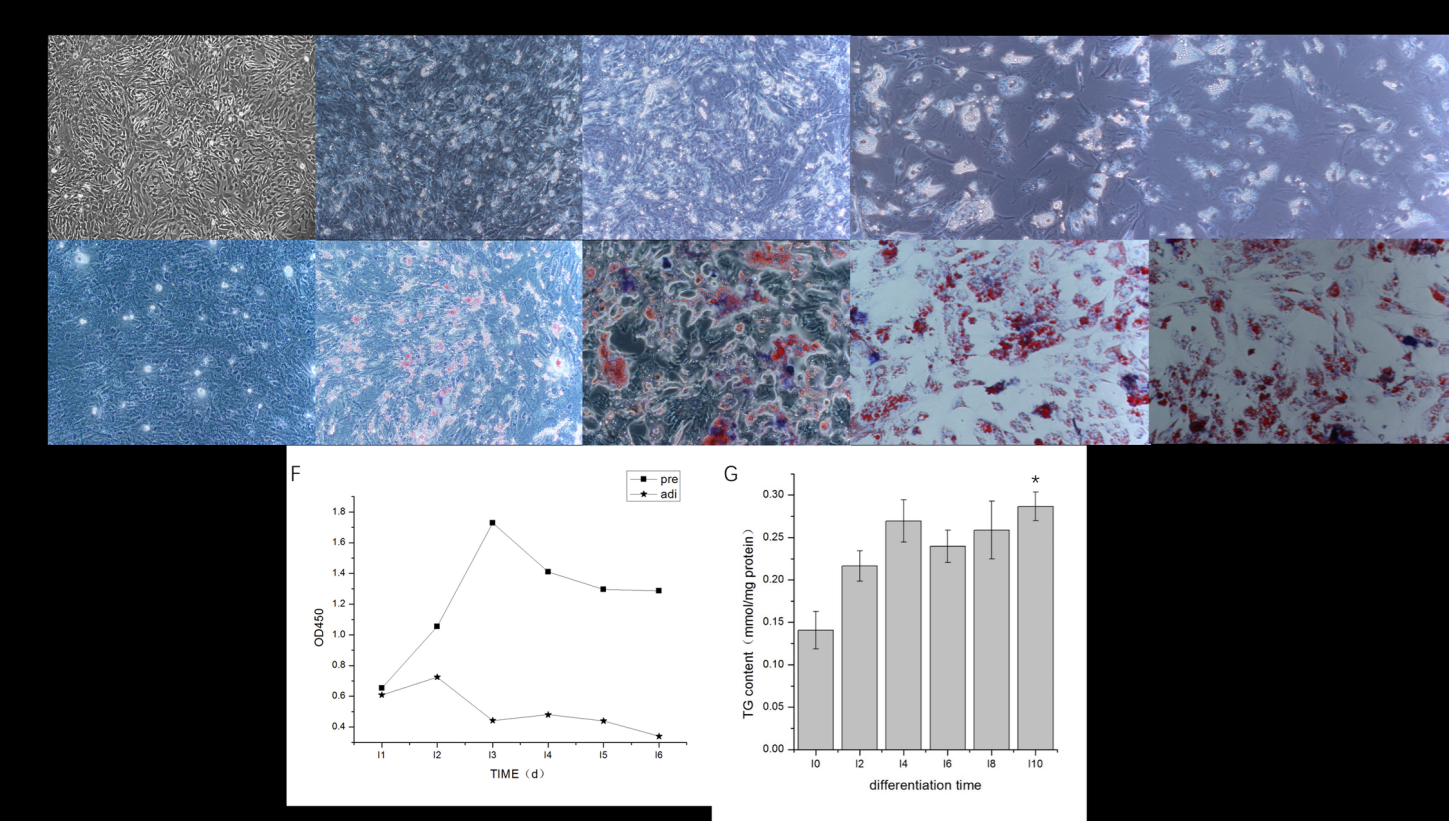
Morphologic characteristics of duck pre-adipocytes at different stages cultured in vitro. Diagrams A, B, C, D, and E represent the morphology of differentiating cells (top row) and staining pattern of Oil Red O (bottom row) under an inverted microscope induced at 0, 2, 4, 6, and 8 days, respectively. Scale bars: 40 μm. F: Growth curve of duck primary and differentiating pre-adipocytes cultured in vitro from day 0 to day 6. G: Changes in the triglyceride content during differentiation at day 0, 2, 4, 6, 8, and 10. Data are presented as the mean ± SD.

Morphological characteristics of adipogenesis in cultured duck pre-adipocytes during differentiation are shown in Fig 1. Pre-adipocytes obtained after collagenase digestion that were adhered to the bottom of the flasks reached 100% confluency after 24 h in the basal culture medium (Fig 1A). Morphology was irregular, like a spindle and triangle. The Oil Red O stain showed no TG formation at this time. After 2 days in the complete induction medium, there were a few small lipid droplets like bright spots in the cells (Fig 1B), and the Oil Red O staining showed some formation of TG. At 4 to 6 days after induction, the number of lipid droplets increased and their volume increased from small to large, some lipid droplets were fused, and there were lipid droplets and exfoliated cells floating in the culture medium (Fig 1C and 1D). At this time, most of the cells were stained red. At 8 days after induction, pre-adipocytes had developed into oval or round mature adipocytes. Lipid droplets became larger and filled the cells, and the nucleus was located at one side instead of in the center (Fig 1E). Almost all the cells were stained red. Actually, the lipid droplets formed a large accumulation of lipid droplet in cells. However, adipocytes, especially those differentiated from primary pre-adipocytes, detached and floated away from the bottom of the flask easily at this stage.

Pre-adipocytes showed an "S-type” growth curve in line with primary cell growth laws (Fig 1F). Cells entered the logarithmic phase during days 2 to 3. Thereafter, pre-adipocytes were in a slow, proliferative state (plateau phase) during which their activities remained stable. Pre-adipocytes essentially stopped proliferating when they started to differentiate into adipocytes. During the differentiation process, the TG content steadily increased (Fig 1G). The content of TG was significantly higher at 10 days after induction compared with that in non-induced pre-adipocytes.

### Expression of genes related to adipogenesis during differentiation

**Fig 2:**
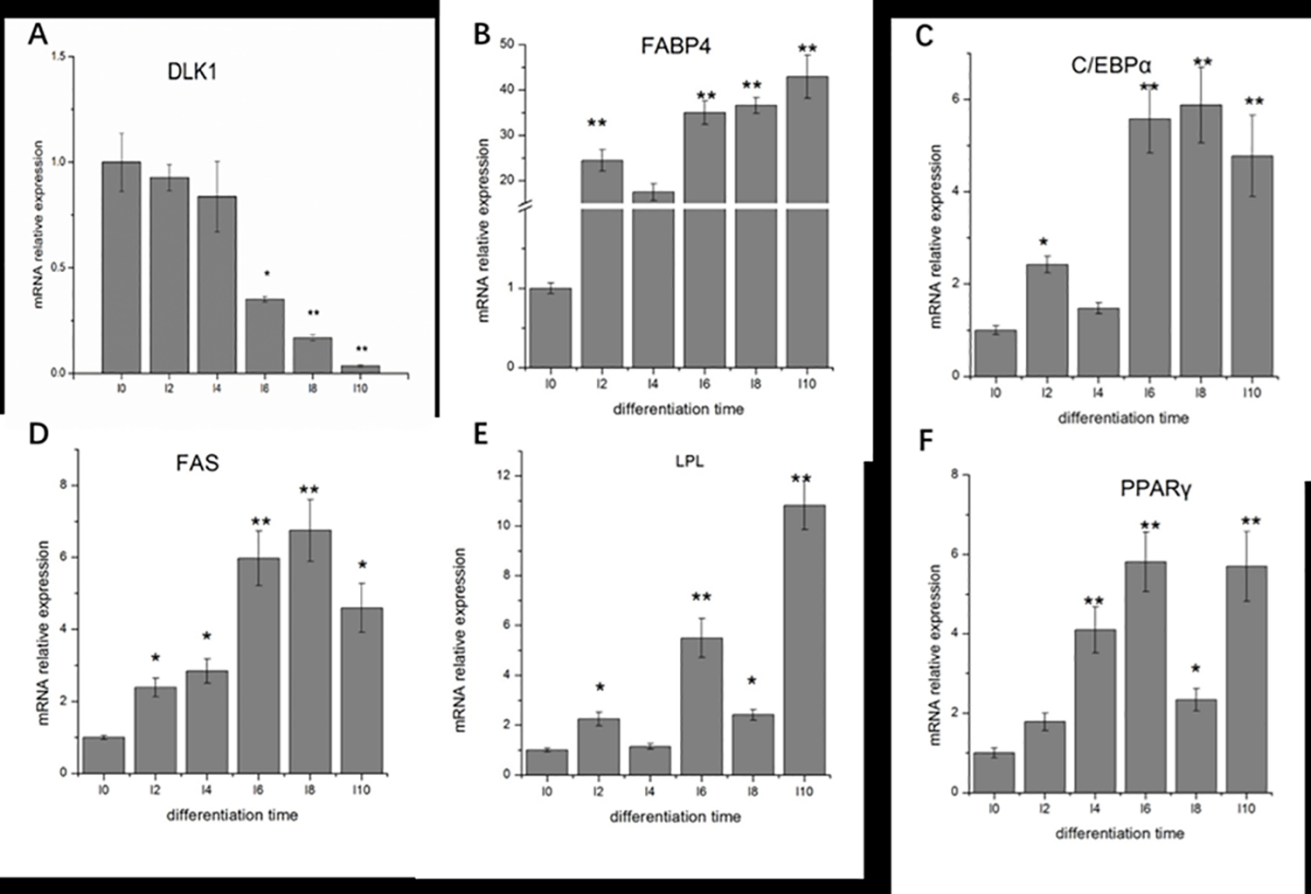
The relative mRNA expression of six genes related to adipogenesis in pre-adipocyte differentiation in duck. A, expression pattern of *DLK1* mRNA; B: expression pattern of *FABP4* mRNA; C: expression pattern of *C/EBPα* mRNA; D: expression pattern of *FAS* mRNA; E: expression pattern of *LPL* mRNA; F: expression pattern of *PPARγ* mRNA during the differentiation of pre-adipocytes. The mRNA expression level at day 0 was assigned as the control. (*) *P* <0.05, (**) *P* <0.01.

The expression profiles of six genes related to adipogenesis during pre-adipocyte differentiation in ducks are shown in Fig 2. All the genes (*DLK1*, *LPL*, *C/EBPα*, *PPAR-γ*, *FAS*, and *FABP4*) were expressed in the cells during pre-adipocyte differentiation and had different expression patterns. The expression of *DLK1* was the highest in pre-adipocytes and decreased gradually during the induction process. The expression of *DLK1* reached its lowest point at 10 days after induction compared with pre-adipocytes (I0) significantly (*P* <0.01). Both *C/EBPα* and *FAS* showed an increased expression pattern during adipogenesis until 8 days after induction, and then decreased at 10 days. *FABP4* increased throughout the differentiation process until day 10 and had the highest level of expression (the highest fold-change was 41.7 at I10). *LPL* showed an increase-decrease expression pattern during adipogenesis and increased to its highest level on day 10, which was significantly higher than that on other culture days (*P* <0.01). *PPARγ* maintained a steady growth rate, except for a clear low peak on day 8 (*P* <0.05).

### The role of oleic acid in adipogenesis

**Fig 3:**
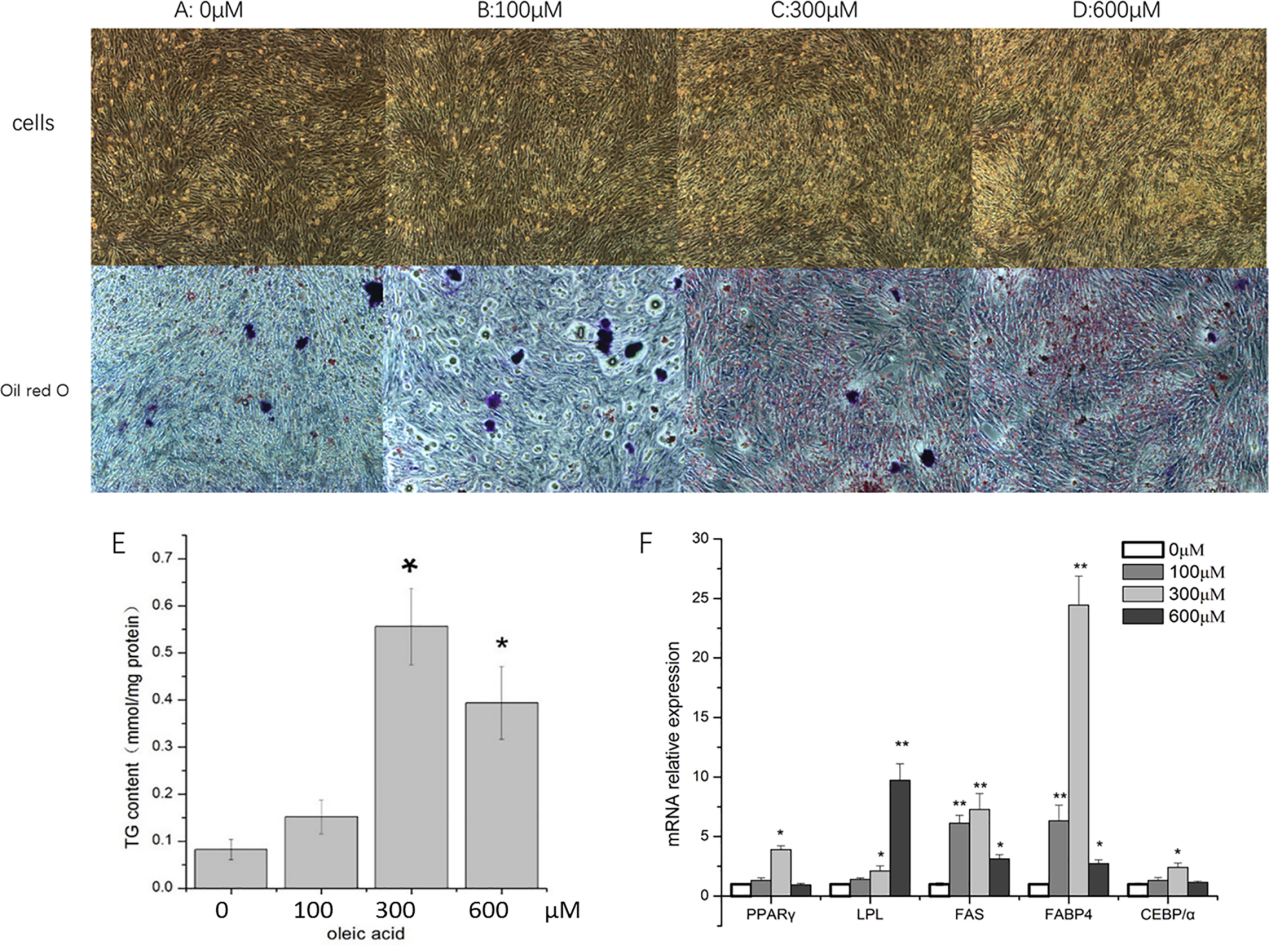
The role of oleic acid in adipogenesis. Morphology of differentiating cells (top row) and staining patterns using Oil Red O (bottom row) under an inverted microscope induced at 3 days by oleic acid at concentrations of: A: 0 μM; B: 100 μM; C: 300 μM; and D: 600 μM. Scale bars: 40 μm. E: change in TG content at different concentrations of oleic acid. F: relative mRNA expression of five genes in relation to concentrations of oleic acid. The mRNA expression level at 0 μM oleic acid was used as the control. Data are presented as mean ± SD. (*) *P* <0.05, (**) *P* <0.01.

We used induction formulas with different concentrations of oleic acid to explore its role in the adipogenesis of pre-adipocytes under 48 h induction. Morphological observations showed little or no lipid droplet formation without oleic acid (Fig 3A). As the concentration of oleic acid increased from 100 μM to 600 μM, the amount of fat deposition also increased (Fig 3B, 3C and 3D). The fatty deposition was at its highest when the oleic acid concentration was 300 μM (Fig 3C). There was no significant increase or decrease in lipid droplets at a concentration of 600 μM oleic acid (Fig 3D) compared with that at 300 μM. Quantitative measurement of TG content were used to evaluate the effect of different oleic acid concentrations on adipocyte adipogenesis. The results were basically consistent with morphological observations (Fig 3E). When oleic acid concentration was 300 μM, TG content was significantly higher than without oleic acid (*P* <0.05).

We examined the expression of five key fat-forming genes at different concentrations of oleic acid (Fig 3F). Except for *LPL*, almost all genes reached their highest peak of expression at 300 μM oleic acid. Therefore, the optimal amount of oleic acid to induce adipogenesis in poultry pre-adipocytes was 300μM.

### Ability to differentiate in different genetic backgrounds

**Fig 4:**
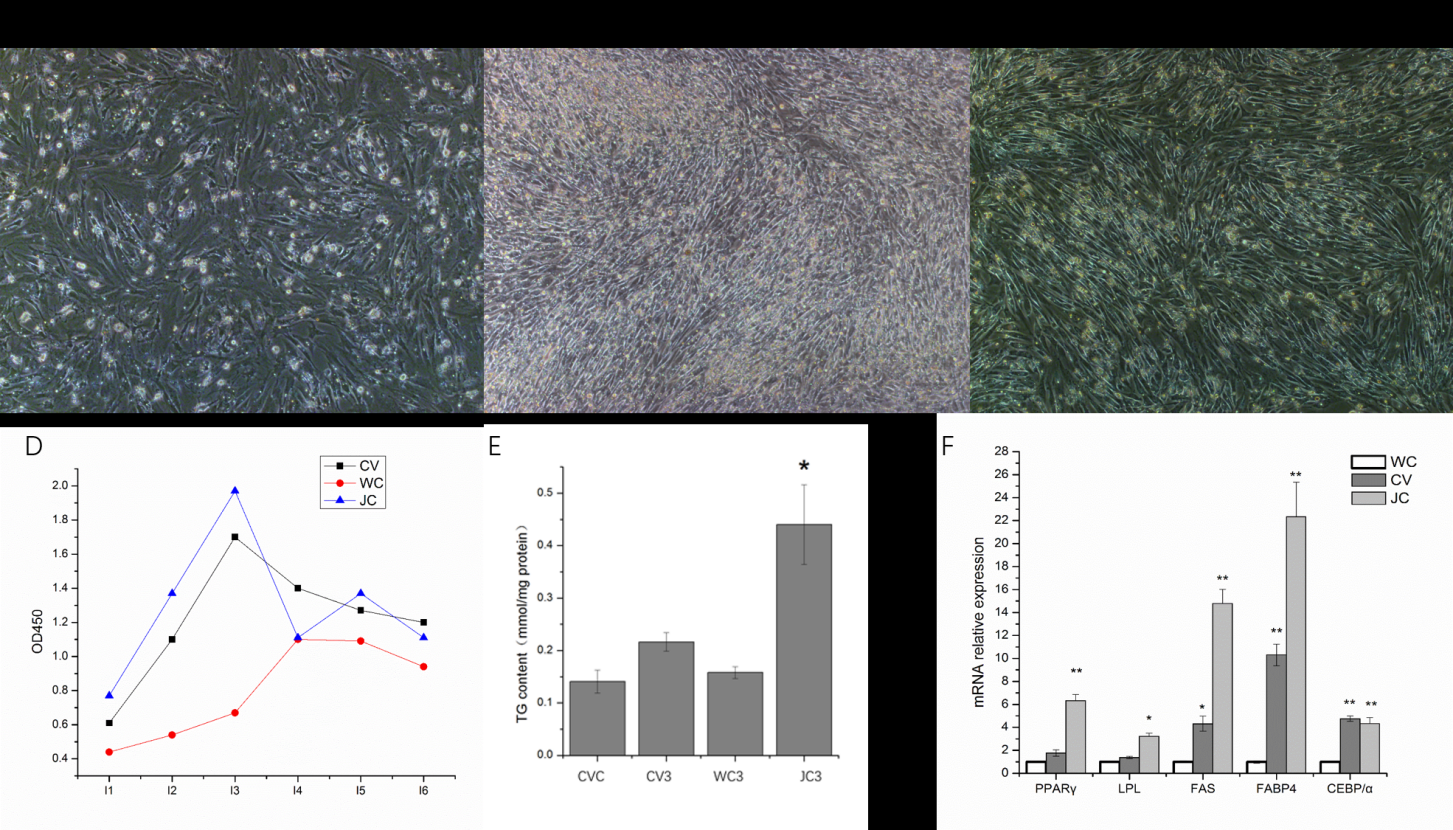
Comparison of the adipogenic ability of different breeds of duck. A: White-crested duck (WC); B: Cherry Valley duck (CV); C: Jianchang duck (JC) at 3 days after induction. Scale bars: 40 μm. D: Comparison of growth curves of pre-adipocytes from WC, CV, and JC. E: Change in TG content between WC, CV, and JC. F: relative mRNA expression of five genes related to the genetic background of each breed. The mRNA expression of WC was used as the control. Data are presented as mean ± SD. (*) *P* <0.05, (**) *P* <0.01.

Morphological observations were performed on pre-adipocytes from three duck breeds: Cherry Valley duck (CV), White-crested duck (WC), and Jianchang duck (JC) at 3 days after induction. As shown in Fig 4, CV and JC produced more lipid droplets from pre-adipocytes than WC. The TG content of adipocytes from each of the three breeds was compared at 3 days after induction (Fig 4D). The pre-adipocytes from CV were used as a control. The TG content in JC was the highest. The proliferating cells were in the logarithmic phase from 24 h to 72 h in all three varieties. We found that JC had the highest proliferative capacity, followed by CV, while WC had the lowest proliferation of pre-adipocytes.

At 3 days after induction, there was a significant difference in the expression levels of the five genes in the three ducks, with JC showing the highest expression in almost all genes, except *C/EBPα*.

### Effect of *PPARγ* interference on adipogenesis

**Fig 5:**
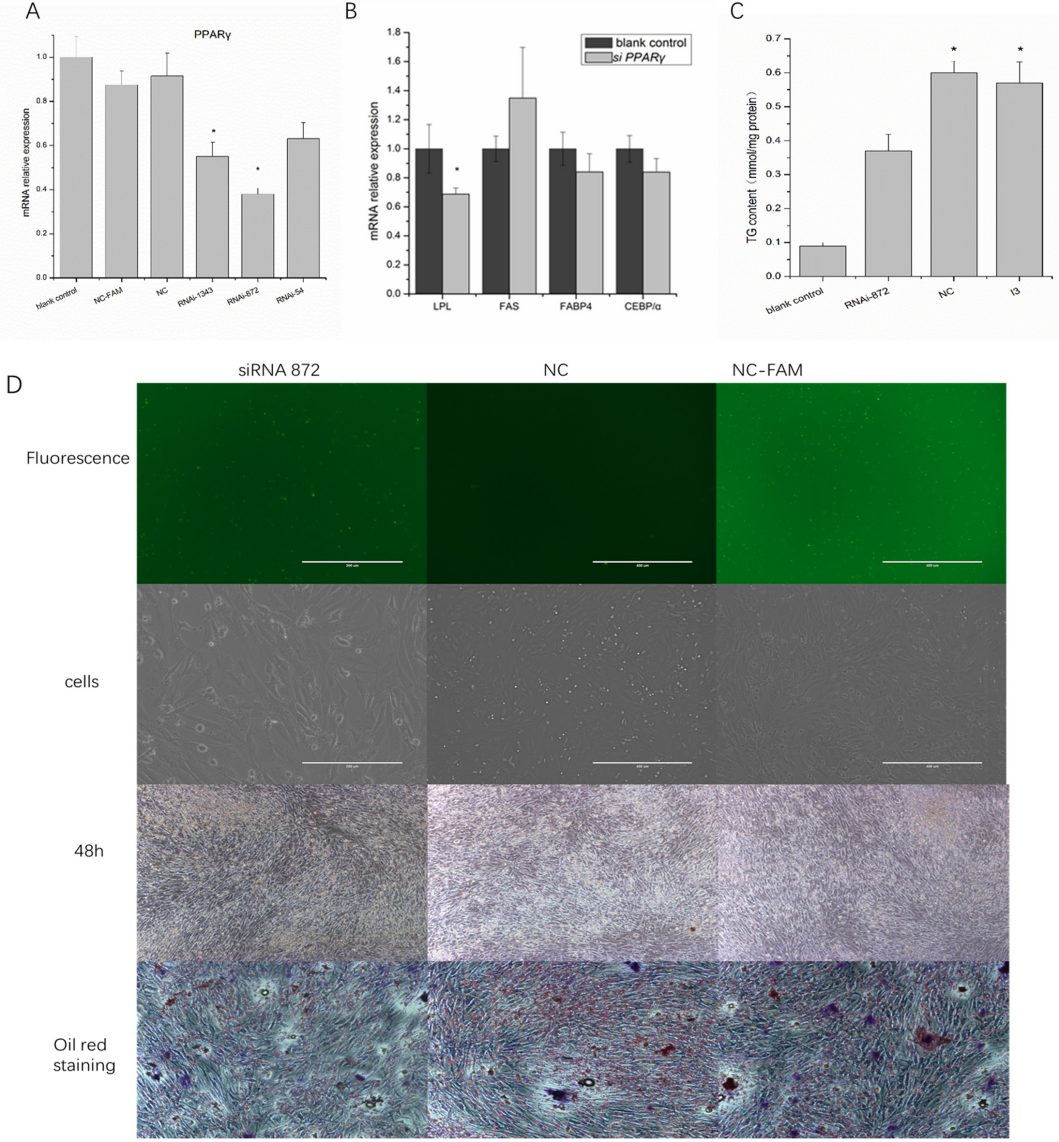
Effect of *PPARγ* interference on pre-adipocytes. A: comparison of three siRNA interference efficiencies. The NC group and NC-FAM were used as fluorescence controls. The blank control was pre-adipocytes without anything added. B: The effect of *PPARγ* interference on expression of related genes. The mRNA expression level of the blank control was assigned as a control. Data are presented as mean ± SD. C: Effect of *PPARγ* interference on TG content. (*) *P* <0.05, (**) *P* <0.01. D: fluorescence and bright field images after 6 h of interference, adipogenic morphology of pre-adipocytes, and Oil Red O staining after 48 h of induction during *PPARγ* interference. Scale bars: 40 μm. NC: negative control. NC-FAM: negative control-Fluorescent marker.

To investigate the role of *PPARγ* in adipogenesis, we performed knock-down experiments using siRNA specific to *PPARγ* (RNAi-1343, RNAi-872, and RNAi-54). Pre-adipocytes were transfected with the siRNAs and cultured for 48 h. We found that siRNA-872 significantly suppressed the expression of *PPARγ* mRNA (Fig 5A) and the highest interference efficiency. We used RNAi-872 to continue the interference of *PPARγ*. After 48 h of interference, the changes in expression of another adipogenic differentiation transcription factor-*C/EBPα* and other genes related to adipocyte differentiation, including *FABP4*, *LPL*, and *FAS*, were detected using qRT-PCR (Fig 5B). The expression levels of *C/EBPα*, *FABP4*, and *LPL* were all decreased compared with the control (induction without interference), and the expression of *LPL* was significantly decreased (*P*<0.05) after the expression of *PPARγ* was decreased. However, the expression level of *FAS* did not change significantly. Quantitative measurements of TG content were used to evaluate the impact of *PPARγ* interference on adipocyte adipogenesis, and showed that TG levels were lower after *PPARγ* interference compared to the control group (I3) (Fig 5C).

Morphological observation and Oil Red O staining showed that *PPARγ* siRNA-872 had almost no effect on adiposity after 48 h induction (Fig 5D), perhaps as part of *PPARγ* was still working, even in the knock-down experiment. As the differentiation progressed, the expression of *PPARγ* gradually increased and played a vital role in adipogenesis.

## Discussion

So far, there have been few systematic studies on the isolation, culture, and induction of duck primary fat cells. In 2012, He et al. [20] reported the functions of *A-FABP* and its regulatory genes related to adipogenesis in duck primary pre-adipocytes. Subsequently, Ding et al. [3] reported a more comprehensive and systematic method for the isolation and induction of duck pre-adipocytes. In this study, we successfully isolated and obtained pre-adipocytes in 20-day-old embryos using these methods. During the induction process, we added rosiglitazone, which differed from the previous methods[21]. At the beginning of induction, the pre-adipocytes gradually shrank from a fusiform shape and became round. The lipid droplets in the cells formed and small lipid droplets gradually accumulated into large lipid droplets. The lipid droplet filled the cytoplasm and it became a mature white adipocyte. This process was similar to that in pre-adipocytes from chicken, which share similar morphologies with primary pre-adipocytes in ducks [12]. The lipid droplets eventually formed large lipid droplets in cells; however, adipocytes that were differentiated from primary pre-adipocytes easily detached from the bottom of the flask at this stage and floated as dead cells.

The expression of *DLK1* in adipocytes is highly specific. We observed that it was highly expressed in pre-adipocytes, but gradually disappeared as the adipocytes differentiated, and it was almost absent in mature adipocytes. A previous study has shown that transfection of constitutive expression components of *Pref-1* significantly reduced the degree of 3T3-L1 adipocyte differentiation [22]. The expression levels of *FABP4*, *C/EBPα*, *FAS*, and *LPL* increased with induction, and maintained a high level of expression compared with pre-adipocytes.

Monounsaturated fatty acids regulate the proliferation and differentiation of pre-adipocytes. Unlike in mammals, poultry adipocytes do not synthesize fatty acids, so extra fatty acids must be added to induce adipogenesis. Previously, it was reported that a concentration of 0.1 mmol L-1 oleate induced the expression of transcriptional differentiation factors, including *PPARγ* and *SREBP-1*, while high levels of oleic acid reduced their expression [23]. In this study, the TG content decreased at 600 μM oleic acid concentration, compared to 300 μM, indicating that high concentrations of oleic acid induced duck adipocyte differentiation, which was consistent with observations of mammalian pre-adipocytes. Thus, high concentrations of oleic acid may have inhibitory effects on fat production.

The number and size of adipose cells determines the amount of adipose tissue[24], so the results for adipocytes in different breeds of duck were inconsistent. Wang et al.[25] made a comparison between the fat and lean broiler lines, a total of 33 miRNAs were found to be significantly differentially expressed, and the target genes of these differentially expressed miRNAs were enriched in pathways related to adipocyte development and lipid metabolism, such as transcription regulation, response to insulin stimulation, and more interestingly, and IGF-1 signalling pathway. Different genetic backgrounds of the same species have different patterns of fat deposition[26]. In our study, we found that CV duck and JC duck had a high amount of adipose tissue, and their pre-adipocyte content and growth rate were faster than WC duck. After induction, the TG content in adipocytes from WC duck was significantly lower than that in JC duck.

*PPARγ* is the most adipocyte-specific and adipogenic member of the PPAR family. It is mainly expressed in adipose tissue and plays an important role in lipid metabolism and adipocyte differentiation [27]. This study explored the role of *PPARγ* in adipogenesis by interfering with *PPARγ*. After the expression of *PPARγ* was knocked-down, the adipogenic capacity was reduced, although not significantly. This could be explained by the decrease in TG content after gene interference. Previous interference experiments with chicken *PPARs* also showed that *PPARγ* inhibits chicken pre-adipocyte proliferation and promotes differentiation [28]. *PPARγ* was at the center of this regulatory network and played an essential role in adipocyte differentiation. In the absence of *PPARγ*, pre-adipocyte differentiation did not begin properly [29]. The interference of *PPARγ* regulated the expression of other fat-related genes, which significantly reduced the expression of *LPL* and had a slight effect on the expression of *FABP4*, *C/EBPα*, and *FAS*, albeit not significantly.
